# Application of multivariate discriminant analysis for differentiation between Saudi sheep (*Ovis aries*) breeds based on physical and histochemical meat characteristics

**DOI:** 10.14202/vetworld.2022.2665-2672

**Published:** 2022-11-22

**Authors:** Gamaleldin M. Suliman, Raed Mahmoud Al-Atiyat, Khaled H. Abu-Alruz, Amer M. Mamkagh, Firas A. Al-Zyoud, Abdullah N. Al-Owaimer, Faisal A. Alshamiry

**Affiliations:** 1 Department of Animal Production, College of Food and Agricultural Sciences, King Saud University, P.O. Box 2460, 11451 Riyadh, Saudi Arabia; 2 Genetics and Biotechnology, Department of Animal Science, Agriculture Faculty, Mutah University, Karak, Jordan; 3 Department of Nutrition and Food Processing, Agriculture Faculty, Mutah University, Karak, 61710, Jordan; 4 Department of Plant Production, Faculty of Agriculture, Mutah University, Karak, 61710, Jordan; 5 Department of Plant Protection and IPM, Faculty of Agriculture, Mutah University, Karak, 61710, Jordan

**Keywords:** discriminant, histochemical, multivariate, sheep meat, traceability

## Abstract

**Background and Aim:**

The multivariate discriminant (MVD) analysis was a successful statistical tool with a discriminatory capacity for tracing sheep breeds based on meat characteristics. Thus, this study aimed to identify three Saudi sheep breeds based on the physical and histochemical aspects of meat using MVD analysis.

**Materials and Methods:**

Eight male lambs from each breed, Najdi, Neami, and Harri, were selected randomly at 90 days of age and allocated into three groups for breeding in a completely randomized design. The feeding and rearing management were similar for an experimental period of 90 days. The experimental diet consisted of a concentrated mixture with identical amounts of calories and nitrogen. Fifty-one meat characteristics were measured in the preliminary MVD, representing hot and cold carcass weight, meat cuts and quality measures, body component weights, fat deposit weights, and histochemical characteristics.

**Results:**

Out of the total meat characteristics measured, only 19 characteristics had significant discriminant power. The most powerful characteristics were temperature, empty intestinal weight, pH_24_, external carcass length, heart weight, and L1, based on partial R-square and Wilks’ lambda values. The phenotypic associations between the characteristics had strong associations. The obtained principal components efficiently classified the eight individuals of each breed into distinct groups using robust discriminant characteristics.

**Conclusion:**

This method allowed us to determine the breed of sheep carcasses and cuts by considering the physical characteristics of the meat. Therefore, butchers and consumers should use scientific techniques for assigning carcasses and meat to their sheep breed after slaughtering.

## Introduction

Local sheep meat markets in developing countries lack traceability regulations and laws [1]. First, sheep meat is mainly provided to these markets without identifying the breed or strain origin. Sheep meat is preferred in the Mediterranean and Gulf regions. This leads breeders, traders, butchers, and consumers to consider its quality and quantity characteristics. However, meat markets have no regulations for trading meat based on grading scale or breed origin. Second, the traceability system in these markets is based on stamp systems authorized by official butcher shops. However, the ability to identify breeds in the system is still lacking. Sheep meat, carcasses, and cuts have different consumer perceptions concerning the breeds they come from. Some consumers prefer the meat of specific breeds for traditional dishes. This is true in the Mediterranean and Gulf regions. Particularly, Saudi Arabian consumers solely select their traditional main dishes to their different local sheep breeds. For example, traditional Saudi dishes called Kabsa, Tharıd, and Jalamah are lamb meat specialties traditionally prepared in different regions using local sheep breeds and consist of unique cuts cooked with their fat.

The differentiation of carcasses and cuts into their sheep breeds of origin has not been previously reported for Saudi sheep. It has been examined in reports how Saudi sheep individuals can be assigned to their breeds while they are still alive. Thus far, butchers and consumers do not possess scientific techniques capable of assigning sheep carcasses to breeds after slaughtering. Many researchers have stated that comparisons between carcass measurements of breeds can be successful, considering factors such as slaughter body weights, ages, and feeding systems [2–5]. These researchers used statistical methods based on quantitative, qualitative, and behavioral characteristics that provide reliable racial discriminants. For instance, simple statistical techniques, such as correlations between breeds when reporting carcass characteristics, have been applied in these studies.

The most successful statistical tool is a multivariate discriminant analysis of meat characteristics, confirming the discriminatory capacity for tracing sheep breeds [6, 7]. This study aimed to identify and trace sheep breeds based on the physical and histochemical characteristics of meat using multivariate discriminant analysis.

## Materials and Methods

### Ethical approval

All the samples used for this study and sampling process were officially issued by the Scientific Research Ethics Committee (SREC) at King Saud University. The animal handling, slaughtering, and sampling were in full agreement with the regulations and protocols nominated by SREC. The study was approved by the Scientific Research Ethics Committee (Approval no. KSU-SE-20-17).

### Study period and location

The study was conducted from 9^th^ March to 10^th^ May, 2017.The experiments were conducted at the experimental farm of the Department of Animal Production, College of Food and Agricultural Sciences at King Saud University, Riyadh, Saudi Arabia (24.8051° N, 46.5203° E). The experiments were conducted during the summer (May, June, and July) when temperatures ranged from 70–110°F (21-44°C).

### Experimental design, animals, and housing

Twenty-four intact male lambs from three main Saudi sheep breeds (eight from each breed) were used in this study. The breeds included Najdi (NJ), Neami (NM), and Harri (HA). The animals were evenly distributed into three groups in a completely randomized design (CRD) in an environmentally controlled barn. The sample size was determined based on similar previous studies that dealt with small animal performance and resulted in significant differences [8, 9]. The size of the samples in this study was determined using the G*Power program (https://www.psychologie.hhu.de/arbeitsgruppen/allgemeine-psychologie-und-arbeitspsychologie/gpower) concerning previous studies that had resulted in significant differences. The eight lambs per breed were recommended to eliminate any possible variation other than breed. Furthermore, this number is not that far from the recommendations made by someone of five lambs. The animals were 3 months old and weighed 15 ± 1 kg.

All animals were ear-tagged and dewormed against internal and external parasites. The feeding period lasted 90 days, preceded by a 14-day adaptation period during which alfalfa (*Medicago sativa*) hay and a graded amount of the experimental diet were provided to the lambs. An isocaloric and isonitrogenous (balanced energy/protein) concentrate mixture was formulated from local feed ingredients available in the market to satisfy all the nutrient requirements of the animals, according to Salah *et al*. [10] (Supplementary Table-S1). The daily ration was offered twice daily at 8:00 am and 3:00 pm. The drinking water and saltlicks were available at all times.

### Carcass, physical, and histochemical characteristics

At the end of the feeding period, all 24 lambs were slaughtered to evaluate their carcasses and their physical and histochemical characteristics. All slaughter data were documented to test the hypothesis of this study by tracing and identifying sheep breeds of meat carcasses, cuts, quality, and histochemical characteristics in the market. A total of 51 characteristics are reported in Supplementary Table-S2. We considered only the significant characteristics from the related statistical analyses (Table-1) and excluded the nonsignificant characteristics. There were 19 significant characteristics in total: Cold carcass weight, body component weight, and fat deposit weight.

**Table-1:** Significance level, means, and standard error of mean of live body and carcass traits for the studied sheep breeds.

Variable	p-value	Breed					

		Harri		Najdi		Neami	
		
		Mean	SEM	Mean	SEM*	Mean	SEM
Cold carcass weight (kg)	0.026	19.22	0.43	20.93	0.43	20.54	0.43
Head weight (kg)	0.013	1.59	0.06	1.87	0.06	1.82	0.06
Heart weight (kg)	0.028	0.14	0.01	0.17	0.01	0.17	0.01
Lungs and trachea (kg)	0.003	0.42	0.03	0.54	0.03	0.55	0.03
Kidney weight (kg)	0.002	0.10	0.00	0.12	0.00	0.12	0.00
Tail weight (kg)	0.002	3.04	0.18	3.01	0.18	3.94	0.18
Empty intestinal weight (kg)	<0.0001	1.01	0.06	1.34	0.06	1.51	0.06
Back fat (cm)	0.001	1.99	0.21	1.29	0.21	2.58	0.21
pH_1_ (Initial pH) (-)	0.038	5.98	0.06	5.91	0.06	6.14	0.06
pH_24_ (Ultimate pH) (-)	0.001	5.87	0.04	6.06	0.04	5.87	0.04
Body temperature (°C)	<0.0001	19.35	0.15	19.69	0.15	18.29	0.15
L_1_* (Initial color lightness) (-)	0.027	27.50	0.94	31.32	0.94	30.10	0.94
Internal carcass length (cm)	0.007	63.38	0.95	67.88	0.95	64.13	0.95
External carcass length (cm)	0.004	61.31	1.13	67.25	1.13	63.13	1.13
Carcass width (cm)	0.049	29.75	0.49	31.56	0.49	30.63	0.49
Rump width (cm)	0.033	40.31	0.63	41.31	0.63	42.81	0.63
Leg length (cm)	0.001	37.31	0.56	39.94	0.56	36.56	0.56
Bone weight (kg)	0.001	23.42	0.91	28.94	0.91	25.52	0.91
Sarcomere length (µm)	0.022	1.79	0.05	1.63	0.05	1.85	0.05

* SEM=Standard error of the mean

In addition, the initial pH (pH_i_) of the meat, temperature, and color values were taken at 1 h postmortem. The final pH (pH_u_) and chill shrink were taken overnight (24 h), chilling at 2°C. The pH and temperature were measured directly in the muscle immediately after slaughter using a microprocessor pH meter (Model PH 211, Hanna^®^ Instruments; Woonsocket, Rhode Island, USA). Two readings were taken, and the mean value was calculated for each carcass. Then, linear carcass measurements were recorded after chilling the carcasses at 2°C for 24 h before cutting them up. The measurements were made with a plastic measuring tape. These measurements included carcass length and carcass width. Carcass fat depth and body wall thickness were measured using a digital Vernier caliper (Model 62065-40, EMS^®^; Electron Microscopy Sciences, Hartfield, PA 19440, USA). The rib-eye area (REA) was measured on the *longissimus thoracis* muscle between the 12^th^ and 13^th^ ribs. An acetate paper was scrabbled over the rib eye and then taken off. The wet paper was then traced using a pen and the paper was left to dry. Then, the area was colored in black and scanned (150 dpi res.) as a monochrome image, which was later analyzed [11] for REA using an analyzing digital image software program (AreaScan 2 MFC Application 2000). Next, a rack cut (6 ribs) was separated to perform carcass dissection. The cut was dissected into meat, bone, fat, and trimmings.

After that, the *longissimus dorsi* (LD) muscle was separated and used to evaluate the proximate chemical composition, using meat quality parameters including cooking loss percentage, drip loss, and water-holding capacity (WHC), as described by Wilhelm *et al*. [12]. In brief, two replicates of about 2 g were collected from the LD muscle of each sample and cut into cubes. Then, the sample was placed between two filter papers and two Plexiglas sheets and set under a 10 kg weight for 5 min. After that, the sample was weighed, and the WHC was determined as the difference between the initial and final weights. The shear force was determined using a modified method outlined by Wheeler *et al*. [13], where modifications included cooking, coring, and shearing of the steaks on the same day, with a 1 h time lapse between cooking and coring.

Texture profile analysis, including the hardness, cohesiveness, springiness, and chewiness variables, was performed using a texture analyzer (TA.HD-Stable Micro Systems^®^; Vienna Court, Lammas Road Godalming Surrey GU7 1YL, UK) equipped with a compression-plate attachment. Each sample underwent two cycles of 80% compression, and the crosshead speed was set at 200 mm/min. A myofibril fragmentation index (MFI) was performed, as described by Wang *et al*. [14]. Shortly after that, a 4 g muscle sample was scissor-minced and then homogenized (Ultra Turrax; Daigger Scientific Inc., Vernon Hills, Illinois, USA) with 40 mL of a cold-isolating MFI buffer. The absorbance of the 0.5 mg/mL solution was determined at 540 nm, and the MFI was determined by multiplying the absorbance value by the dilution factor. The sarcomere length (SAR) was determined by following the methods of Cross *et al*. [15]. Finally, a subjective evaluation test was performed.

### Statistical multivariate discriminant analysis

Data in the form of both continuous quantitative variables were collected and constructed into a simple discriminant analysis (SAS) format file. The data were then subjected to discriminant and clustering analyses using SAS version 9.2 [16]. SAS statistical analyses were performed to calculate means (PROC MEANS), general linear model, and mean separation test of least differences as a mean separation procedure, along with SAS DISCRIM to calculate the probability of identifying lamb by its predefined breed. In addition, the stepwise discriminant procedure (STEPDISC) was applied to determine which body-measured traits would be used in the final clustering analysis. Another type of procedure, canonical discriminant analysis (CANDISC), was used to perform uni- and multivariate analysis to derive the canonical variables (CAN) for the best match of the breed with lamb meat [17]. Furthermore, genetic square distances (Mahalanobis distances) were also generated. These distances were used to construct a dendrogram using MEGA software 5.0 (https://www.megasoftware.net/) [18].

## Results and Discussion

The statistical descriptions of the studied phenotypic traits for lamb breeds are shown in Table-1. Nineteen traits had a significant effect on breeds. Their mean and standard error values are presented in Table-1. The table shows higher values for the NJ breed, except for a few characteristics, such as tail and back fat weight and rump width, in NM lambs. The characteristics were analyzed to determine their association with each other (Table-2) as a first step before performing the stepwise selection statistical analysis.

The significant correlation coefficients in Table-2 significantly correlated between cold carcass weight and all characteristics except pH_24_, temperature, initial color lightness (L_1_*), bone weight, SAR, and L. Similarly, head weight was significantly correlated with all characteristics except pH_24_, temperature, and L1. In general, the physical characteristics of the meat were more highly correlated with one another compared to the histochemical characteristics. These physical characteristics have economic importance for consumers in the differentiation of slaughtered animals [19]. Consequently, the high phenotypic correlation between most of the studied physical characteristics of the carcass would be efficient in the study of breed differentiation.

**Table-2:** Pearson correlation coefficients of live body and carcass traits for the studied sheep breeds.

Variable	Head	Heart	L and T	Kidneys	Intestine empty	pH 24	Temp	L1	Internal carcass length	External carcass length	Carcass width	Carcass rump width	Leg length	Bone	SAR and L
Cold carcass	0.62**	0.60**	0.60**	0.62**	0.60**				0.56**	0.76¤	0.55*	0.53*	0.51*		
Head		0.48	0.63**	0.61**	0.61**				0.60**	0.67**	0.54*	0.46^+^	0.47^+^	0.43^+^	
Heart			0.69**	0.45^+^	0.40^+^				0.44^+^	0.45^+^	0.45^+^	0.44^+^			
Lungs and trachea				0.63**	0.66**				0.47^+^	0.56**	0.48^+^	0.50*		0.58**	
Kidneys					0.73¤				0.49^+^	0.63**		0.41^+^		0.44^+^	
Tail							-0.63**					0.56**			
Empty intestine									0.42^+^	0.45^+^		0.55*			
Back fat						−0.53*	−0.45^+^		−0.52*				−0.44^+^		0.46^+^
pH_1_							−0.48^+^	−0.45^+^					−0.54^+^		
pH_24_														0.42^+^	−0.70¤
Carcass temperature												−0.47^+^	0.42^+^		
L_1_														0.45^+^	
Internal carcass Length										0.69**	0.45^+^		0.69**	0.57*	
External carcass Length											0.64**	0.42^+^	0.77¤	0.51*	
Carcass width													0.69**	0.43^+^	
Leg length														0.53*	

^+^p < 0.05,

* p < 0.01,

** p < 0.00, ¤p < 0.0001. L_1_: initial color lightness. SAR=The sarcomere length

The univariate procedure within the multivariate discriminant analysis for providing the significant discriminate power (p < 0.05) of the characteristics is shown in Table-3. All 19 characteristics of physical and histochemical types showed power in discriminating lamb carcass breeds based on estimated variation values, R-square, F-test values, and level of significance (p < 0.05). The most efficient characteristics for discriminating lamb breeds were carcass temperature, empty intestinal weight, pH_24_, external carcass length, heart weight, and L1. They were ranked in the previous order as a result of the high average squared canonical correlation (p < 0.0001) and higher R-square, Wilks lambda, and F values compared to the other studied traits (Table-4). On the other hand, eigenvalues had a very high value (45.231) in canonical function 1 (CAN1), reaching 80.2% of the total variation of all characteristics in the set (Table-5).

**Table-3:** Univariate test statistics of live body and carcass traits for the studied sheep breeds. (Numerator degrees of freedom (DF)=2, Denominator DF=21).

Variable	Total SD	Pooled SD	Between SD	R-square	R-square	F-value	p > F
Cold carcass weight	1.38	1.21	0.90	0.29	0.42	4.37	0.026
Head weight	0.21	0.18	0.15	0.34	0.51	5.34	0.013
Heart weight	0.03	0.02	0.02	0.29	0.41	4.25	0.028
Lungs and trachea	0.09	0.07	0.07	0.43	0.77	8.05	0.003
Kidney weight	0.02	0.01	0.01	0.45	0.81	8.50	0.002
Tail weight	0.66	0.51	0.53	0.45	0.81	8.46	0.002
Empty intestinal weight	0.27	0.17	0.25	0.63	1.71	17.99	<0.0001
Back fat weight	0.79	0.60	0.65	0.47	0.89	9.36	0.001
pH_1_ (Initial pH)	0.19	0.17	0.12	0.27	0.36	3.83	0.038
pH_24_ (Ultimate pH)	0.13	0.10	0.11	0.49	0.96	10.04	0.001
Carcass temperature	0.73	0.42	0.73	0.69	2.23	23.43	<0.0001
L_1_* (initial color lightness)	3.02	2.66	1.95	0.29	0.41	4.33	0.027
Internal carcass length	3.26	2.69	2.41	0.38	0.61	6.44	0.007
External carcass length	3.96	3.19	3.04	0.41	0.69	7.29	0.004
Carcass width	1.51	1.37	0.91	0.25	0.33	3.49	0.049
Rump width	1.99	1.77	1.26	0.28	0.38	4.04	0.033
Leg length	2.12	1.59	1.77	0.49	0.95	9.97	0.001
Bone weight	3.39	2.58	2.79	0.47	0.89	9.32	0.001
Sarcomere length	0.18	0.15	0.12	0.31	0.44	4.61	0.022

SD=Standard deviation

**Table-4:** Stepwise selection summary of discriminant power of live body and carcass traits.

Variable	R-square	F-value	Pr > F	Wilks’ Lambda	Pr < Lambda	Canonical correlation	Pr > ASCC
Carcass temperature	0.69	23.43	<0.0001	0.31	<0.0001	0.35	<0.0001
Empty intestinal weight	0.61	15.92	<0.0001	0.12	<0.0001	0.60	<0.0001
pH_24_	0.50	9.38	0.002	0.06	<0.0001	0.75	<0.0001
External carcass length	0.47	7.94	0.003	0.03	<0.0001	0.81	<0.0001
Heart weight	0.23	2.52	0.110	0.02	<0.0001	0.84	<0.0001
L_1_*	0.25	2.64	0.102	0.02	<0.0001	0.86	<0.0001

**Table-5:** Function, eigenvalue, variance percentage, and canonical correlation.

CAN	Canonical correlation	Adjusted canonical correlation	Approximate standard error	Squared canonical correlation	Eigenvalue	Difference	Proportion	Cumulative
1	0.9891	0.9802	0.0045	0.9784	45.231	34.046	0.802	0.8017
2	0.9581	0.9294	0.0171	0.9179	11.185		0.1983	1

CAN=Canonical

**Figure-1:**
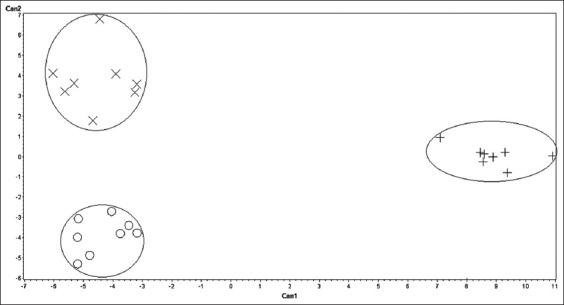
Canonical representation (CAN1 and CAN2) of principal component analysis of the three Saudi lamb breeds where Neami is represented by (+), Najdi is represented by (X), and Harri is represented by (O) using the most discriminating traits. The discriminating traits of the highest power in assigning the lambs into their breed in CAN1 were, as an example, tail weight, intestine empty weight, back fat weight, and pH1 and thus their differentiating power plotted along X-axis. They were, in CAN2, bone weight, external carcass length, pH24, kidneys weight, internal carcass length, head weight, L and T, L1, intestine empty weight, cold carcass weight, leg length, and carcass width plotted along Y-axis.

The canonical functions CAN 1 and 2 assigned lamb characteristics to their functions as the percentage of correct assignment (Table-6). The most discriminating traits in CAN 1 were tail weight, empty intestinal weight, back fat weight, and pH_1_ coefficient >0.5. It was shown in the results that those characteristics had the highest power in assigning the lambs to their possible breeds, and thus, they were differentiated along the X-axis (Figure-1). The most discriminating traits in CAN 2 were bone weight, external carcass length, pH_24_, kidney weight, internal carcass length, head weight, L, and T, L1, empty intestinal weight, cold carcass weight, leg length, and carcass width (Table-6). Their discriminant power was presented along the Y-axis of the principal component analysis in Figure-1. It was revealed in the results that those traits had the highest loading (coefficient > 0.5).

**Table-6:** Total canonical structure: the CANDISC procedure.

Variable	Can1	Can2	Can3	Can4
Tail weight	0.6753	−0.012	0.0271	−0.038
Empty intestinal weight	0.6037	0.5472	−0.137	−0.052
Back fat weight	0.5883	−0.38	0.0838	−0.073
pH_1_	0.5034	−0.144	0.1499	0.0707
Rump width	0.486	0.2252	0.077	0.0039
Sarcomere length	0.3989	−0.403	0.146	−0.004
Lungs and trachea	0.3782	0.566	0.001	−0.13
Kidney weight	0.3303	0.6092	−0.082	0.022
Heart weight	0.2651	0.489	0.0415	−0.049
Head weight	0.1961	0.5713	−0.04	−0.127
Cold carcass weight	0.1548	0.5426	0.0461	−0.07
L_1_*(Initial color lightness)	0.1047	0.5535	0.1166	−0.128
Carcass width	−0.017	0.5208	−0.019	−0.146
Bone weight	−0.104	0.7076	−0.103	−0.113
External carcass length	−0.15	0.6499	0.0358	−0.011
Internal carcass length	−0.232	0.5975	−0.078	−0.067
pH_24_ (Ultimate pH)	−0.351	0.6332	0.0055	−0.232
Leg length	−0.481	0.533	0.0405	−0.062
Carcass temperature	−0.82	0.1901	−0.194	0.122

CANDISC=Canonical discriminant analysis

It has been reported in previous studies that the loading value of the structure matrix shows the correlation of each variable with each discriminant function [20–22]. The highest loading of traits suggests that the correlation between them is the function that discriminates between the individuals in the discriminant function. The discriminant relationship of all studied lamb carcasses was better presented in the multiple correspondence analyses (Figure-1) with the data obtained from the canonical discriminant analysis. The structure of this relationship is shown in Table-6. It is clear from the figure that sheep carcasses were separated from each other and formed three distinct groups. The groups were predefined breeds: NM, NJ, and HA. NJ and HA were closer to one another, while broad discrimination could be seen between these and NM carcasses. This result was expected considering the similar genetic origins of NJ and HA. On the other hand, we would expect the discrimination power of the major discriminating trait to show less variation between NJ and HA. It is worth mentioning that a smaller distance can be seen between the carcasses of each breed. In other words, the multiple correspondence analyses classified the carcasses into separate clusters representing their predefined breeds: NM, NJ, and HA.

**Figure-2:**
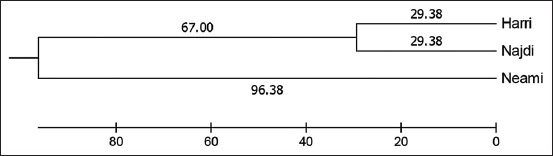
Dendrogram showing evolutionary genetic tree of the three Saudi lamb breeds, Harri, Najdi, and Neami, based on live body and carcass measurements. The tree was plotted using the UPGMA method based on the significant genetic distances (Mahalanobis distances) (p < 0.0001) between the breeds as in Figure-2. The evolutionary history indicates one cluster had both Harri and Najdi breeds with the sum of branch length = 222.14 (96.38 + 67.00 + 29.39 + 29.38) unit. The Najdi breed was being located in the intermediate position reflecting close evolutionary history to Harri with the sum of branch length = 58.76 (29.38 + 29.38). The evolutionary history of optimal tree between Neami and Harri breeds was estimated of branch length =192.76 (96.38 + 67.00 + 29.38) indication longer distance.

These results are better shown by showing the genetic distances (Mahalanobis distances) between the breeds (Figure-2). An evolutionary genetic tree, as a genetic distance between breeds, with branch lengths in the same units as those of the evolutionary distances used to infer the phylogenetic tree, is shown in Figure-2 [18, 21]. The distances between all pairs were significant (p < 0.0001). The evolutionary history was inferred using the Unweighted Pair Group Method with Arithmetic Mean (UPGMA). The optimal tree, which had a branch length sum of 222.14, is shown. The tree is drawn to scale, with branch lengths in the same units as the evolutionary distances used to infer the phylogenetic tree. The sheep evolutionary history inferred using the UPGMA method is shown in Figure-2, in which one cluster had both HA and NJ breeds. The NJ breed was located in the intermediate position, reflecting its close evolutionary history to HA in the optimal tree, with a branch length sum of 58.175. The evolutionary history of the optimal tree between NM and HA breeds had an estimated branch length of 190.70, indicating a longer distance. Overall, the breeds of live animals and their carcasses were successfully identified by canonical discriminant analysis based on the above mentioned 13 traits.

## Conclusion

In this study, the sheep carcasses of three Saudi sheep breeds were assigned and traced based on the physical and histochemical characteristics of the meat. As previously reported by many researchers, multivariate discriminant analysis was very successful in the assignment test. Although many carcass traits are considered in the national market, only limited characteristics had significant discriminant power in the analysis. Therefore, it is essential to consider the most significant and powerful features when tracing the breed of sheep carcasses. The eight individuals were efficiently clustered by these characteristics into their distinct breeds by using principal component analysis. In summary, we recommend that the findings of this study be considered when tracing the breed of meat sheep according to the physical and histochemical characteristics of meat.

## Data Availability

The data generated during the study are available within the published article.

## Authors’ Contributions

GMS, RMA, ANA, and FaAA: Experimental design, execution, and data collection. GMS, RMA, and KHA: Data analysis and writing the manuscript. GMS, RMA, KHA, AMM, and FiAA: Research concept. All authors have read and approved the final manuscript.

## Supplementary Tables

**Table-S1:** Ration composition of the isocaloric and isonitrogenous (balanced energy/protein) concentrate mixture.

Item	DM basis (%)
Ingredients	
Alfalfa hay	25.0
Maize	39.3
Barley	23.8
Soybean meal	9.1
Mineral supplement*	2.6
Premix**	0.2
Chemical composition	
CP	13.50
CF	8.16
ADF	15.0
NDF	49.42
NFE	67.77
EE	2.74
Ash	8.60
Ca	0.56
P	0.29
ME Mcal/kg***	2.73

* 30% sodium bicarbonate, 30% ground limestone, 20% dicalcium phosphate, 20% chloride.

** CoSO_4_ (0.30 g), CuSO_4_ (20.1 g), FeSO_4_ (10.0 g), ZnO (50.0 g), MnSO_4_ (40.2 g), KI (0.75 g), NaCl (2.81 g), Vitamin A (500,000 IU), Vitamin D (500,000 IU), and Vitamin E (10,000 IU).

*** Calculated

**Table-S2:** Non-significant characteristics (32) excluded from the statistical analyses.

Variable	R- square	F value	Pr > F
Body organs			
Leg weight	0.02	0.20	0.824
Liver weight	0.16	1.90	0.176
Spleen weight	0.16	1.97	0.166
Stomach empty weight	0.28	3.96	0.055
Gut fill weight	0.12	1.33	0.287
Wholesale cuts			
Shoulder weight	0.02	0.21	0.813
Rack weight	0.14	1.58	0.231
Loin weight	0.13	1.45	0.259
Foreshank and breast weight	0.02	0.24	0.789
Dissection components			
Meat weight	0.00	0.02	0.978
Fat weight	0.05	0.47	0.629
Trimmings weight	0.13	1.47	0.253
Color components			
L2* (Ultimate color lightness)	0.09	0.99	0.391
a1* (Initial color redness)	0.17	1.99	0.163
a2* (Ultimate color redness)	0.02	0.19	0.832
b1* (Initial color yellowness)	0.20	2.51	0.107
b2* (Ultimate color yellowness)	0.01	0.09	0.913
Texture profile analysis			
Chewiness	0.06	0.63	0.545
Cohesiveness	0.14	1.60	0.226
Hardness	0.06	0.67	0.525
Springiness	0.03	0.27	0.769
Meat physicochemical traits			
Cooking loss	0.07	0.71	0.505
Drip loss	0.07	0.74	0.489
Chill shrinkage	0.13	1.43	0.263
Myofibril fragmentation index	0.17	2.08	0.152
Water-holding capacity	0.07	0.74	0.488
Rib-eye area	0.10	1.17	0.331
Fat depots			
Kidney-knob and channel fat weight	0.20	2.56	0.102
Mesenteric fat weight	0.21	2.61	0.098
Omental fat weight	0.01	0.12	0.888
Pericardial fat weight	0.04	0.43	0.655
Body wall fat weight	0.10	1.08	0.359
